# Comparison of rabbit rib defect regeneration with and without graft

**DOI:** 10.1007/s10856-016-5807-1

**Published:** 2016-11-19

**Authors:** Feng-Zhen Liu, Da-Wei Wang, Yu-Jue Zhang, Zhao Yong Lv, Xiao-Dan Sun, Ke-Yi Li, Bin Zhang, Xiu-Mei Wang, Fu-Zhai Cui

**Affiliations:** 1 Liaocheng People’s Hospital, Medical College of Liaocheng University, ShanDong, 252000 China; 2 State Key Laboratory of New Ceramics and Fine Processing, School of Materials Science and Engineering, Tsinghua University, Beijing, 100084 China

## Abstract

Rib segment, as one of the most widely used autologous boneresources for bone repair, is commonly isolated with an empty left in the defect. Although defective rib repair is thought to be unnecessary traditionally, it’s of vital importance actually to promote rib regeneration for patients with better postoperative recovery and higher life quality. Comparative investigations on rabbit rib bone regeneration with and without graft were reported in this article. A segmental defect was performed on the 8th rib of 4-month-old male New Zealand rabbits. The mineralized collagen bone graft (MC) was implanted into the defect and evaluated for up to 12 weeks. The rib bone repair was investigated by using X-ray at 4, 8 and 12 weeks and histological examinations at 12 weeks after surgery, which showed a higher bone remodeling activity in the groups with MC implantation in comparison with blank control group, especially at the early stage of remodeling.

## Introduction

Rib defects are quite common in clinic, which can occur as a result of trauma, thoracotomy, and autologous bone harvest [1–5]. For example, oral and maxillofacial surgeons commonly use autologous ribs to repair the mandibular defects [6] and neurosurgeons also use autologous ribs to repair a large and complex skull defects [7, 8]. In the past, little attention was paid to the rib defect reconstruction as it was always thought to have little impact on patient’s respiratory function. Traditionally, the defective ribs are not treated after being harvested, which actually will lead to a lot of adverse influences, such as chest wall deformities, cardiopulmonary insufficiency problems, and so on so forth [9]. With the development of improved surgical techniques and the increase on patients’ esthetic concerns, rib defect repair has gradually gained more and more attentions. Therefore, it is valuable to investigate the repair and regeneration of rib defects by means of tissue-engineered bone grafts.

To date, few attempts have been made to regenerate rib defect where a completely segmental defect exists and the bioactive bone materials are needed. Recently, mineralized collagen (MC) had been successfully applied in many types of bone repair, which is fabricated by an *in vitro* biomimetic mineralization process and has highly similar chemical compositions and microstructures to the natural bone tissue [10–13]. Collagen fibers in the MC assembled in order and served as the templates for the nucleation and growth of the HA crystals [14]. The effects of the MC on repairing bone defects and promoting new bone regeneration have been demonstrated in many animal models and clinical practices, such as the treatments of intervertebral fusion and bone nonunion [15–19].

The purpose of this work was to investigate the effect of MC bone materials on promoting the rabbit rib defect regeneration. In this work, MC graft was implanted into the rabbit rib bone defect, which was observed for up to 12 weeks, providing experimental evidence for the potential application of MC bone materials in rabbit rib defect regeneration.

## Experimental part

### Ethics and materials

All the animal experiments performed between September and December in 2015 were approved in accordance with regulations set forth by the institution’s animal care and oversight committee, located at Laboratory Animal Center of Liaocheng Food and Drug Supervision Bureau. All surgeries were taken under general anesthesia and all the efforts were made to minimize rabbits suffering. Eight healthy New Zealand white rabbits (age ~150 ± 7 days old, weight ~3.0–3.5 kg) were provided by Laboratory Animal Center of Shandong University [permission No.SCXK (Lu) 20150001]. MC bone materials were provided by the School of Materials Science and Engineering of Tsinghua University [12, 18]. Experimental protocols complied with Regulations for the Administration of Affairs Concerning Experimental Rabbits, formulated by the Ministry of Science and Technology of China.

### Group management and preparation of rib bone defect models

**Fig. 1:**
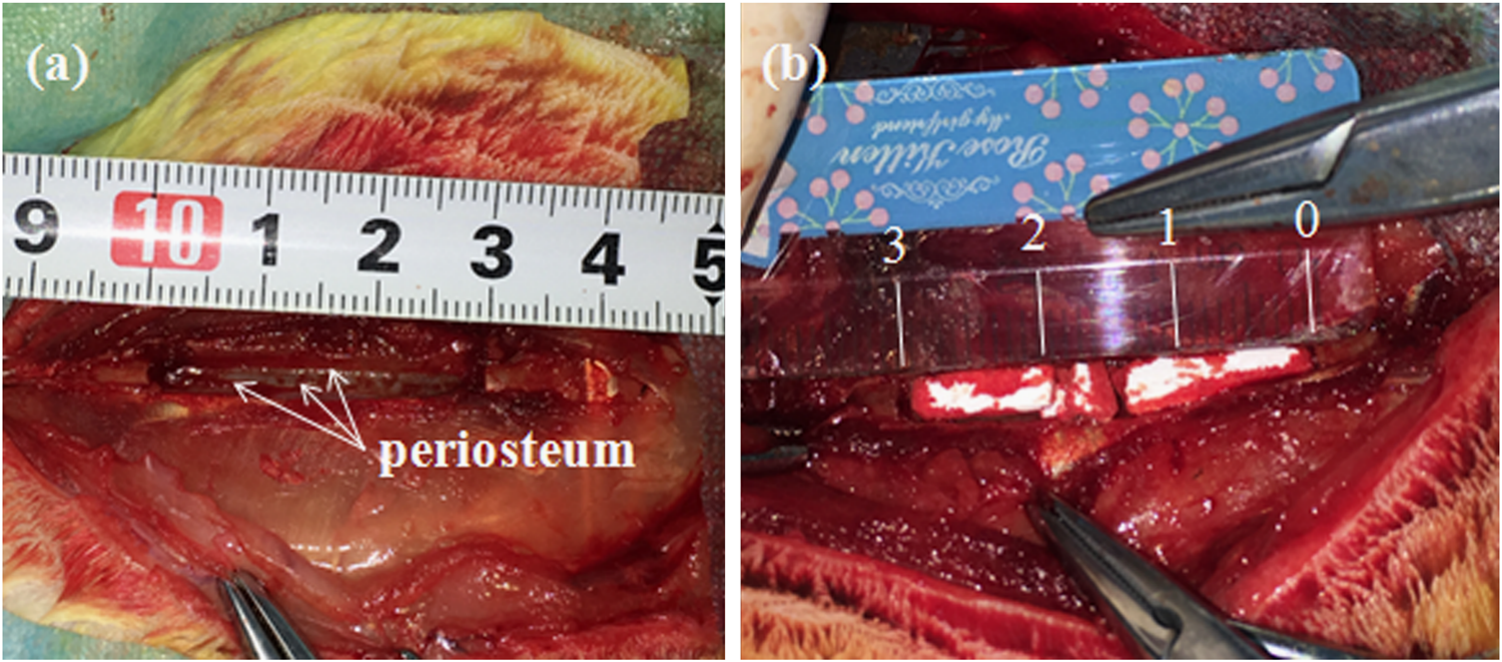
Experimental procedures. **a** Group A: blank control group, the periosteum was preserved (*white arrows*, periosteum on the both sides and bottom), MC was not implanted in the ribdefect. **b** Group B: MC group, MC was implanted in the bone segment (3 cm defect)

Eight healthy New Zealand white rabbits, weighing approximately 300–350 g were housed, one rabbit per cage, in temperature (15–21 °C), air flow and light (12 h day and 12 h night) controlled roomsand received rabbit food and water adlibitum. The rabbits were randomly allocated to two groups, blank control group and MC group. All the surgical procedures were performed under systemic anesthesia using 10 % (vol/vol) chloral hydrate in oxygen for ~2 min. Assessment of the depth of anesthesia is according to the lack of reflex to toe pinch. After anesthetizing, the rabbits were immobilized in a lateral position. The right abdomen was upward and the surgical site was shaved, isolated with sterile drapes, scrubbed with surgical antiseptic, sterilized with alcohol andiodine, and covered with sterile towels. A skin incision was made in the right abdomen, then the subcutaneous fascia and muscles were incised. After exposing the ribs, the incisions were made on the right flank, parallel to rib orientation. An orthopedic ribs scissors was used to remove a section of the ribs and the periosteum on both the lateral margin and bottom was preserved (Fig. 1a). The fracture edges of the rib were smoothed using a rasp to meet the designed size. The MC was implanted into the defect zone and their position was checked (Fig. 1b). The controlled group was not implanted any materials. The rib bone fragments, related coagulation scab, and rib bone marrow tissues were washed with 50 mL normal saline. After hemostasis and wound rewashing, the subcutaneous fascia, muscles and skin incision were sutured with absorbable 4/0 surgical sutures by suturing in two layers after saline irrigation and sterilized with alcohol and iodine. After surgery, the experiment rabbits were injected with penicillin intramuscularly every day for 3 days in succession. The rabbits were housed separately. Rabbits had free access to food and water and were monitored daily in the postoperative period for any complications or abnormal behavior.

### Radiography analysis

Ordinary radiograph examinations of the rabbit ribs were taken in the mediolateral projections(46 kV, 6.0 mA, 10 ms, digital X-ray machine, Siemens AG, Munich, Germany) at 4, 8 and 12 weeks after the operation. The rabbits were anaesthetized and ketamine (40 mg kg^−1^) by intramuscular injection. The rabbits were laid on their backs. Then, photographic film was placed on the right abdomen and X-ray photos were taken at 6 mA, 46 kV, for 10 ms.

### Histological examinations

The rabbits were sacrificed 12 weeks after surgery. The specimens from bone defect sites were fixed in 10 % neutral buffered formalin for 48 h. Then they were fixed with 10 % paraformaldehyde, and were decalcified by 5 % Ethylene Diamine Tetraacetic Acid (EDTA). After decalcification, the samples were cut along the longitudinal plane on the microtome, then embedded in lab-grade paraffin wax. The longitudinal plane sections (parallel to the long axis of bone, 5 mm in thickness) were prepared and stained with hematoxylin and eosin (H&E). Then they were observed using a light microscope (IDA-2000, Konghai Technology and Development Co., Beijing, R. P. China).

## Results

### Mortality and clinical signs

All the rabbits after surgery described above recovered quickly, returned to routine activities such as grooming, eating and drinking within 48 h. No apparent signs of infection such as red, hot incision or exudate were observed. No other test object related clinical signs were observed.

### Radiography analysis

**Fig. 2:**
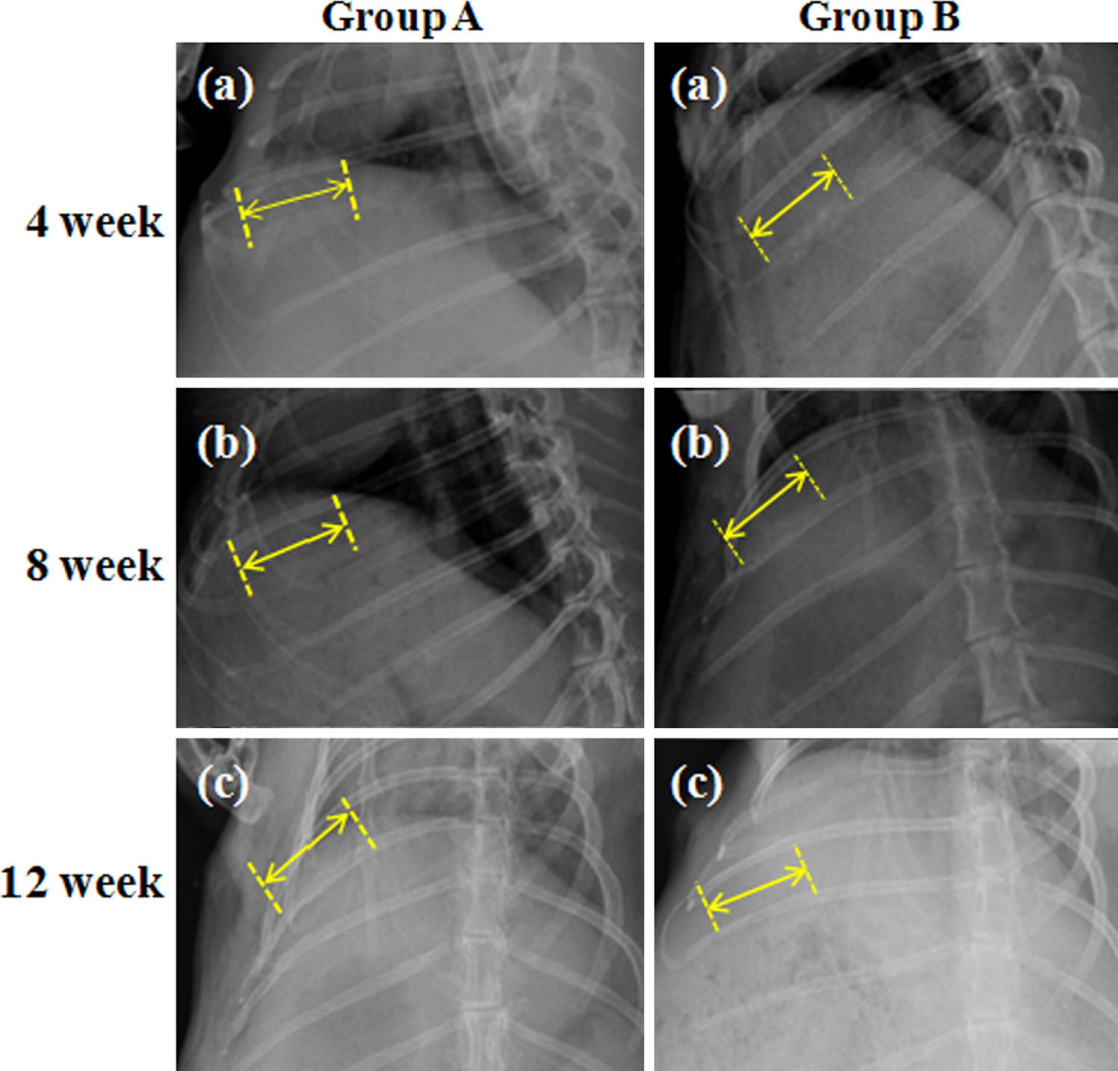
X-ray radiographs of rabbit rib defects at 12 weeks after surgery. Group A: blank control group, MC was not implanted in the rib defect. Group B: MC group, MC was implanted in the bone segment (3 cm defect)

X-ray radiographs showed the 3 cm defects were covered with homogeneous opacity and bone union in the MC group, as shown in Fig. 2 (Group B). It could be clearly seen that a large amount of new bone formed and the defects were nearly fully repaired at 4 weeks postoperatively. Moreover, bone modeling and remodeling had occurred at 8 and 12 weeks after operation. At 12 weeks after surgery the rib defects were covered with newly-formed calluses and the broken ends were connected with a bony bridge. However, in Fig. 2 (the blank control Group A), obvious radiolucency was observed at 4 weeks postoperatively indicating few new bone formation,which implied that the rate of new bone formation in the control group was slower than that of MC group, and the gaps could still be seen at 4 weeks postoperatively.

### Histological examinations

**Fig. 3:**
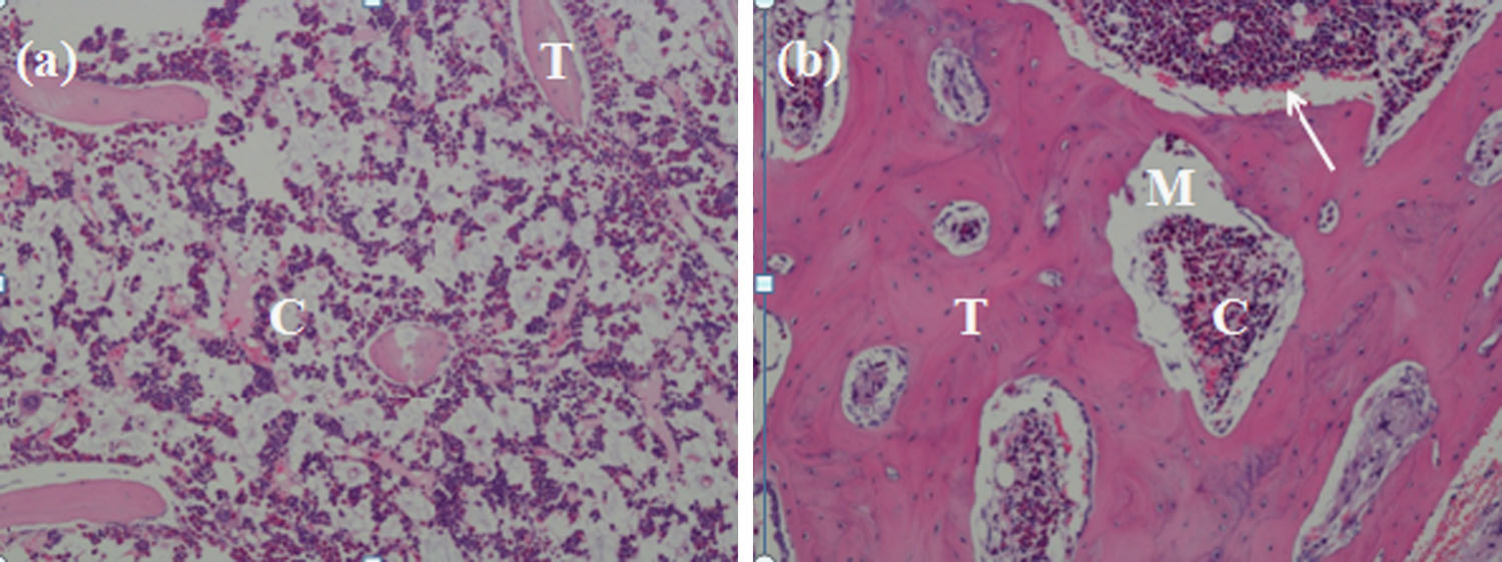
Hematoxylin-eosinphotographs (H&E × 40 magnification) of rabbit rib defects at 12 weeks after surgery. **a** The blank control group, C: osteoblast; T: New bone trabecular, **b** MC group, MC was implanted. M: materials; C: osteoblast; *white arrow*: the newly formed capillary vessel; T: New bone trabecular

The present histological results revealed that relatively mature bone with well-arranged bone trabecula (Fig. 3b, T: new bone trabecular), the growth of blood vessels (Fig. 3b, white arrow: newly formed capillary vessel) andosteogenic cellscould be found around the bone trabecular in the MC group at 12 weeks postoperatively. Haversian system has formed, and the newly formed bone and bone marrow cavity have developed well. However, only few bone trabecular bones were observed in control group (Fig. 3a). These findings suggested that MC exhibited the capacity to promote new bone formation. MC could offer a satisfactory biological environmentfor new bone invasion with in the implants and stimulating new bone deposition. It can act not only as a void filler facilitating guided tissue regeneration, but also as an accelerator for the healing process [20–22].

**Fig. 4:**
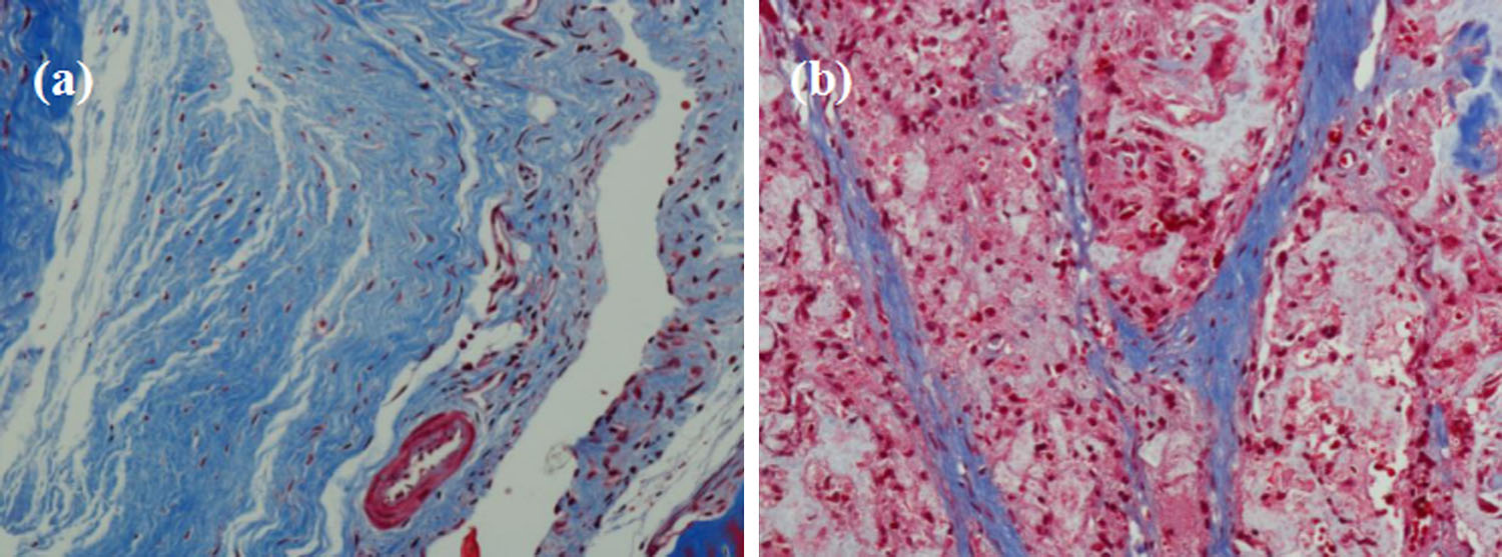
Histology photomicrographsof Masson staining of rabbit rib defectsimplanted with MC at 12 weeks after surgery. **a** *Blue*: collagen of new bone, **b** *Red*: collagen of mature bone, *white*: MC

With the new bone invasion, MC bonematerial degraded gradually and was separated by the new bone tissue into several small islands, as shown in Fig. 4. The blue staining indicated the collagen of newly formed bone, and the red section indicated collagen of mature bone (Figs. 4a, b). After implantation, surrounding bone tissue grew along the porous MC scaffold. These can attribute to the fact that MC is mineralized collagen fibrils, which shows satisfactory biocompatibility at cellular lever and often predominates in the formation of the fibrous capsule that surrounds the implant [23].

## Discussion

In this study, we tried to address the problem-repairof rib bone defects.In the past several years, little research directed towards addressing this problem and no progress has been made. Some special considerations must be made when designing a graft to repair a rib defect. First, the rib has a variable radian and thus a single graft may not be suitable for rib reconstruction as the graft must be easily molded. In this study, we used MC to reconstruct rib bone defect. This method was first reported in this study. We successfully reconstructed a long rib defect with MC. In the present study, a higher bone remodeling activity was observed around the group repaired with MC than that group without MC, especially at the early stage of remodeling, which indicated rib defect reconstruction was important and necessary. The rib is not a load bearing bone but may move with respiration. Although we have achieved a good result with respect to the rib reconstruction, some problems should be studied further. There still, however, remain several problems such as the mechanical integrity of the grafts. Although rib is a bone that suffers very little outer force, because of the effect of the chest muscle, the graft still is exposed to deforming forces, especially when the animal vocalizes. We think that such a technique might be a feasible approach for rib bone repair but further research should be done.

## Conclusion

The results indicated that the defective rib bone rapidly regenerated with MC implantation, showing more mature bone tissue in comparison with the blank control group. This study provided experimental evidence on the feasibility of rib bone defect regeneration using artificial bone graft.

## References

[1] Gupta SS, Singh O, Soni M, Raikwar RS, Mathur RK (2009). Extra-osseous Ewing’s sarcoma of chest wall. ANZ J Surg.

[2] Schwartz GS, Rios L, Zivin-Tutela T, Bhora FY, Connery CP (2009). Uncommon etiology of an anterior chest wall mass. Ann Thorac Surg.

[3] Kridel RW, Ashoori F, Liu ES, Hart CG (2009). Long-term use and follow-up of irradiated homologous costal cartilage grafts in the nose. Arch Facial Plast Surg.

[4] Bapat MR, Chaudhary K, Garg H, Laheri V (2008). Reconstruction of large iliac crest defects after graft harvest using autogenous rib graft: a prospective controlled study. Spine.

[5] Liu FZ, Chen K, Hou L, Li KY, Wang DW, Zhang B, Wang XM (2016). Determining the critical size of a rabbit rib segmental bone defect model. Regen Biomater.

[6] Brian S, Jayanta M, Hanifa AL (2014). 5th rib osteo-pectoralismajor myocutaneous flap-still a viable option for mandibular defect reconstruction. Indian J Otolaryngol Head Neck Surg.

[7] Ichiro T, Masataka A (2008). Catcher’s mask crnioplasy for extensive cranialdefects in children with an open head trauma: a novel application of partial cranioplasty. Childs Nerv Syst.

[8] Arum KS, Devi PM, Vijay K (2011). Spectrum of primary bonegrafting in cranio maxillofacial trauma at a tertiary care centre in India. Indian J Plast Surg.

[9] Antonella L, Simona P, Marianna R (2012). Rib cage deformitiesalter respiratory muscle action and chest wall function in patients with severe osteogenesisimperfecta. PLoS ONE.

[10] Hu NM, Chen ZG, Liu X, Liu HY, Lian XJ, Wang XM, Cui FZ (2012). Mechanical properties and in vitro bioactivity of injectable and self-setting calcium sulfate/nano-HA/collagen bone graft substitute. J Mech Behav Mater.

[11] Wang XM, Cui FZ, Ge J, Wang Y (2004). Hierarchical structural studies of bones from gene-mutated *lilput*^*dtc232*^ Zebrafish. J Struct Biol.

[12] Zhang W, Liao SS, Cui FZ (2003). Hierarchical self-assembly of nano-fibrilin mineralized collagen. Chem Mater.

[13] Kikuchi M, Itoh S, Ichinose S, Shinomiya K, Tanaka J (2001). Self-organization mechanism in a bone-like hydroxyapatite/collagen nanocompositsynthesized in vitro and its biological reaction in vivo. Biomaterials.

[14] Cui FZ, Li Y, Ge J (2007). Self-assembly of mineralized collagen composites. Mater Sci Eng R: Reports.

[15] Qiu ZY, Zhang YQ, Zhang ZQ, Song TX, Cui FZ (2015). Biodegradable mineralized collagen plug for the reconstruction of craniotomy burr-holes: a report of three cases. Transl Neurosci Clin.

[16] Liu X, Wang XM, Chen ZG, Cui FZ, Liu HY, Mao KY, Wang Y (2010). Injectable bone cement based on mineralized collagen. J Biomed Mater Res Part B-Appl Biomat.

[17] Yu X, Xu L, Cui FZ, Qu Y, Lian XJ, Wang XM, Wang Y (2012). Clinical evaluation of mineralized collagen as a bone graft substitute for anterior cervical intersomatic fusion. J Biomater Tissue Eng.

[18] Qiu ZY, Cui Y, Wang XM, Cui FZ (2015). Mineralized collagen: rationale, current status, and clinical applications. Materials.

[19] Lian K, Lu H, Guo XD, Cui FZ, Qiu ZY, Xu SY (2013). The mineralized collagen for the reconstruction of intra-articular calcaneal fractures with trabecular defects. Biomaterials.

[20] Walsh WR, Morberg P, Yu Y, Yang JL, Haggard W, Sheath PC, Svehla M, Bruce WJM (2003). Response of acalcium sulfate bone graft substitute in a confined cancellous defect. Clin Orthop Relat Res.

[21] Li XM, Feng QL, Liu XH, Dong W, Cui FH (2006). Collagen-based implants reinforced by chitin fibres in a goat shank bone defect model. Biomaterials.

[22] Guo BL, Lei B, Li P, Ma PX (2015). Functionalized scaffolds to enhance tissue regeneration. Regen Biomater.

[23] Zheng H, Zhang J, Zhou AG (2006). Experimental study of nano-hydroxyapatite and polyamide composite pin about rigidity of bone-to-pin interface. J Traumatic Surgery.

